# Supplementary figures and images for: Urine proteome changes in rats subcutaneously inoculated with approximately ten tumor cells
Source: PeerJ. 2019 Sep 17;7:e7717. doi: 10.7717/peerj.7717 (PMC6753921; doi:10.7717/peerj.7717)

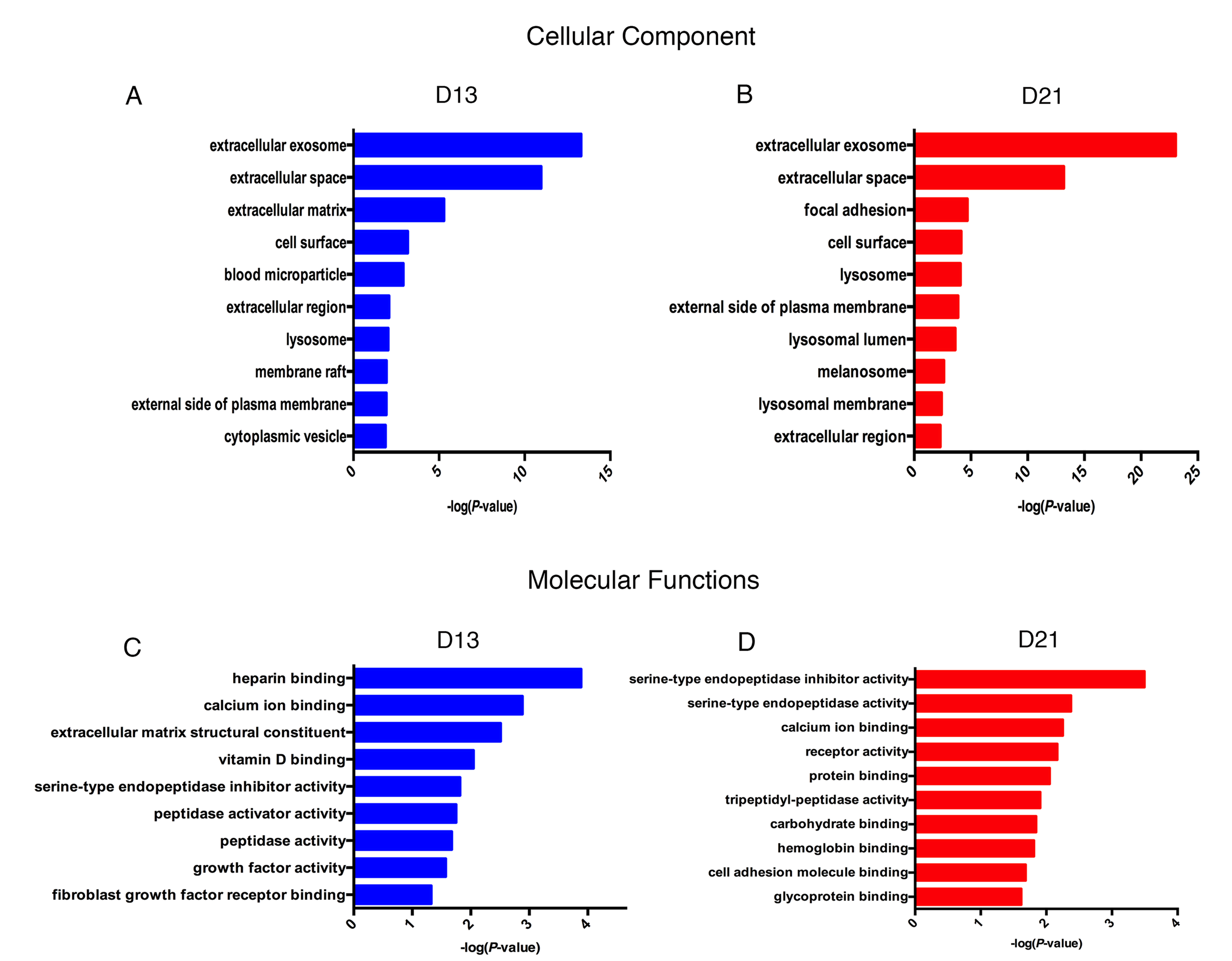

Supplement: Figure S1 — (A) Dynamic changes in cellular component on day 13. (B) Dynamic changes in cellular component on day 21. (C) Dynamic changes in molecular functions on day 13. (D) Dynamic changes in molecular functions on day 21. [file peerj-07-7717-s001.png]

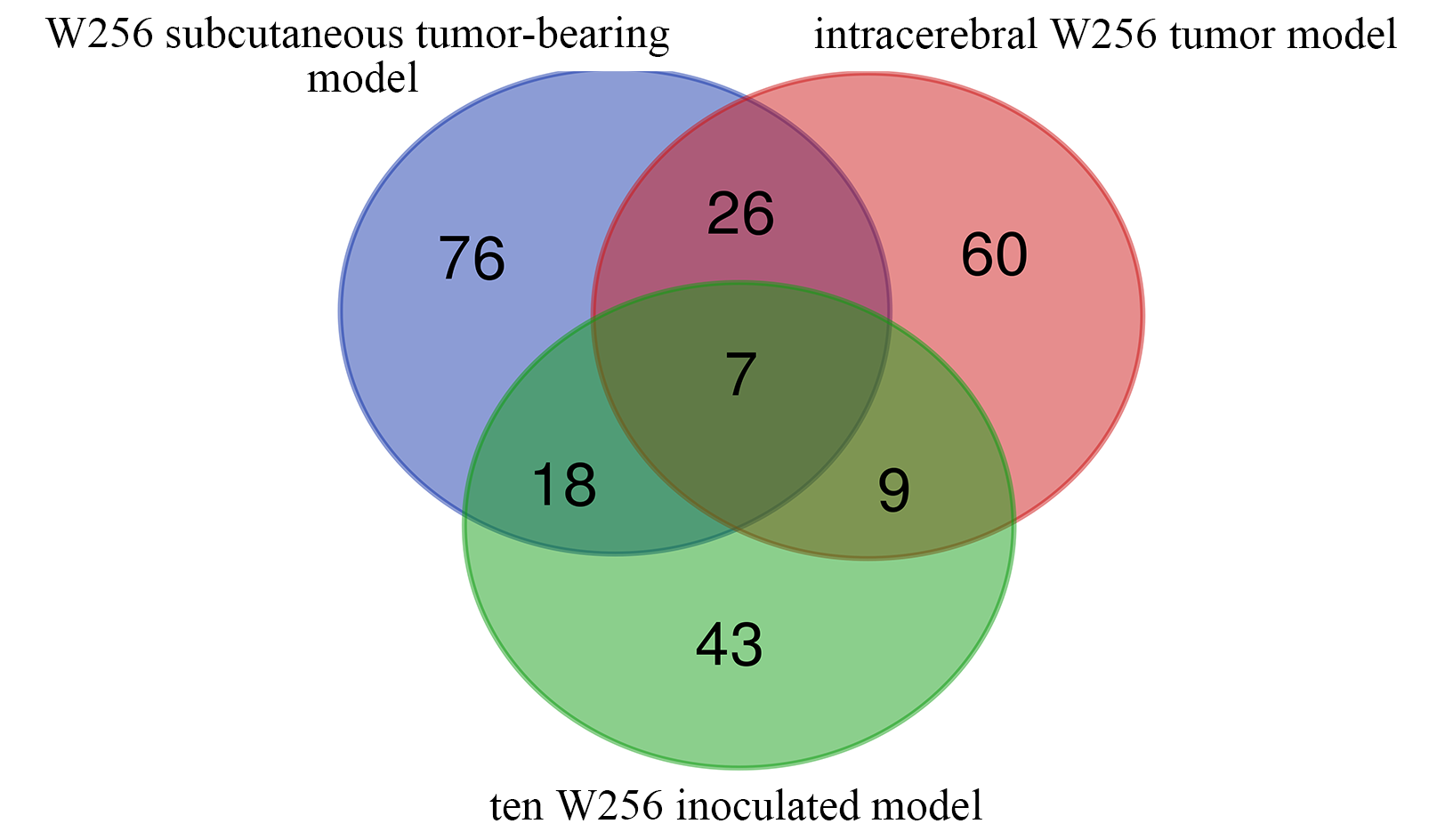

Supplement: Figure S2 [file peerj-07-7717-s002.png]
